# Supplementary material for: Systemic Metabolic and Volumetric Assessment via Whole-Body [18F]FDG-PET/CT: Pancreas Size Predicts Cachexia in Head and Neck Squamous Cell Carcinoma
Source: Cancers (Basel). 2024 Sep 30;16(19):3352. doi: 10.3390/cancers16193352 (PMC11475137; doi:10.3390/cancers16193352)
Supplement: Supplementary file 1 [file cancers-16-03352-s001.zip › cancers-3216636-supplementary.pdf]

cancers

MDPI

Supplementary Materials: Systemic Metabolic and Volumetric Assessment via Whole-Body [18F]FDG-PET/CT: Pancreas Size Predicts Cachexia in Head and Neck Squamous Cell Carcinoma

Josef Yu, Clemens Spielvogel, David Haberl, Zewen Jiang, Öykü Özer, Smilla Pusitz, Barbara Geist, Michael Beyerlein, Iustin Tibu, Erdem Yildiz, Sam Augustine Kandathil, Till Buschhorn, Julia Schnöll, Katarina Kumpf, Ying-Ting Chen, Tingting Wu, Zhaoqi Zhang, Stefan Grünert, Marcus Hacker and Chrysoula Vraka

Table S1. Clinical, Imaging, and Body Composition Characteristics by WL.

		Entire data (N=253)	LoWL (N=107)	HiWL (N=146)	p-Value
Overall Survival		47.74 (35.58)	52.86 (32.89)	44.03 (36.98)	**0.03***
One Year Survival		191 (75.49%)	90 (84.11%)	101 (69.18%)	**< 0.01****
Sex (Male)		198 (78.26%)	85 (79.44%)	113 (77.4%)	0.76
Tumor origin	Oropharynx	172 (67.98%)	78 (72.90%)	93 (63.70%)	0.23
	Larynx	28 (11.07%)	12 (11.21%)	15 (10.27%)	
	Oral Cavity	23 (9.09%)	7 (6.54%)	16 (10.96%)	
	Hypopharynx	21 (8.30%)	5 (4.67%)	16 (10.96%)	
	Nasopharynx	5 (1.98%)	2 (1.87%)	3 (2.05%)	
	CUP	4 (1.58%)	3 (2.80%)	1 (0.68%)	
AJCC 8th	I	8 (3.16%)	4 (3.74%)	4 (2.74%)	0.88
	II	13 (5.14%)	7 (6.54%)	6 (4.11%)	
	III	27 (10.67%)	12 (11.21%)	15 (10.27%)	
	IVa	175 (69.17%)	72 (67.29%)	103 (70.55%)	
	IVb	20 (7.91%)	9 (8.41%)	11 (7.53%)	
	IVc	10 (3.95%)	3 (2.80%)	7 (4.79%)	
HPV	negative	73 (28.85%)	29 (27.1%)	44 (30.14%)	0.30
	positive	35 (13.83%)	18 (16.82%)	17 (11.64%)	
	not reported	145 (57.31%)	60 (56.07%)	85 (58.22%)	
Feeding Tube		131 (51.78%)	47 (43.93%)	84 (57.53%)	**0.04***
Smoking		175 (69.17%)	63 (58.88%)	112 (76.71%)	**0.03***
		Imaging Parameters			
Tumor SUV		6.25 (3.06)	5.92 (2.61)	6.47 (3.3)	**0.04***
Metabolic Tumor Volume		25.19 (31.69)	20.25 (24.74)	28.36 (35.08)	0.06
Adrenal glands SUV		1.73 (0.5)	1.66 (0.49)	1.78 (0.49)	**0.05***
Adrenal glands Volume		8.75 (3.23)	8.94 (3.07)	8.61 (3.33)	0.51
Aorta SUV		1.96 (0.48)	1.94 (0.43)	1.97 (0.5)	0.87
Aorta Volume		262.38 (64.29)	263.89 (61.65)	261.29 (66.12)	0.61
GI tract SUV		1.37 (0.35)	1.36 (0.35)	1.37 (0.34)	0.74
GI tract Volume		2000.92 (577.65)	2106.3 (556.73)	1923.69 (580.49)	**< 0.01****
Heart SUV		2.41 (1.05)	2.43 (1.06)	2.39 (1.04)	0.78
Heart Volume		585.99 (145.09)	603.8 (149.31)	572.95 (140.5)	0.12
Left Atrium SUV		2.13 (0.57)	2.13 (0.52)	2.13 (0.6)	0.83
Left Atrium Volume		81.68 (28.93)	84.37 (32.56)	79.7 (25.77)	0.41
Right Atrium SUV		1.85 (0.48)	1.84 (0.45)	1.85 (0.51)	0.86
Right Atrium Volume		89.83 (24.96)	92.07 (25.92)	88.18 (24.1)	0.25
Myocardium SUV		3.06 (2.4)	3.06 (2.35)	3.05 (2.43)	0.68
Myocardium Volume		131.77 (35.3)	136.39 (36.35)	128.38 (34.11)	0.10
Left Ventricle SUV		2.93 (1.55)	2.98 (1.57)	2.9 (1.53)	0.57
Left Ventricle Volume		120.08 (32.86)	123.87 (34.04)	117.3 (31.69)	0.15
Right Ventricle SUV		1.94 (0.59)	1.96 (0.59)	1.93 (0.59)	0.60
Right Ventricle Volume		162.66 (40.81)	167.09 (41.28)	159.41 (40.15)	0.12
Kidneys SUV		2.72 (0.67)	2.78 (0.83)	2.68 (0.51)	0.68
Kidneys Volume		349.15 (86.3)	356.24 (89.26)	343.95 (83.69)	0.27
Liver SUV		2.29 (0.51)	2.28 (0.46)	2.3 (0.55)	0.78
Liver Volume		1655.09 (428.99)	1726.7 (443.51)	1602.61 (410.16)	**0.02***
Lungs SUV		0.62 (0.17)	0.67 (0.17)	0.58 (0.16)	**< 0.01****

	Entire data (N=253)	LoWL (N=107)	HiWL (N=146)	p-Value
Lungs Volume	3657.74 (975.9)	3492.64 (860.17)	3778.73 (1036.09)	0.05
Pancreas SUV	1.64 (0.43)	1.6 (0.42)	1.68 (0.44)	0.17
Pancreas Volume	75.66 (24.17)	81.34 (22.22)	71.51 (24.69)	**< 0.01****
Pelvis SUV	1.23 (0.33)	1.18 (0.29)	1.26 (0.35)	0.09
Pelvis Volume	1068.77 (179.66)	1083.71 (175.24)	1057.75 (182.07)	0.19
Ribs SUV	0.98 (0.21)	0.97 (0.21)	1.0 (0.21)	0.34
Ribs Volume	440.89 (95.43)	452.06 (94.28)	432.7 (95.43)	0.12
Spinal Cord SUV	1.42 (0.3)	1.42 (0.31)	1.42 (0.3)	0.98
Spinal Cord Volume	95.41 (27.6)	96.28 (24.54)	94.77 (29.65)	0.34
Spleen SUV	1.78 (0.37)	1.76 (0.35)	1.8 (0.38)	0.45
Spleen Volume	238.42 (114.0)	264.78 (124.32)	218.97 (101.44)	**< 0.01****
Vertebra SUV	1.58 (0.38)	1.53 (0.35)	1.61 (0.4)	0.18
Vertebra Volume	834.52 (148.03)	839.28 (151.24)	831.04 (145.54)	0.72
SKM (WB) SUV	0.82 (0.26)	0.8 (0.31)	0.83 (0.21)	**0.01***
SKM (WB) Volume	9538.65 (2896.76)	9647.2 (3035.91)	9458.05 (2786.22)	0.38
SAT (WB) SUV	0.54 (0.24)	0.52 (0.28)	0.55 (0.21)	**0.01***
SAT (WB) Volume	14438.1 (8408.81)	16428.36 (8317.15)	12960.49 (8168.36)	**< 0.01****
TAT (WB) SUV	0.96 (0.23)	0.97 (0.24)	0.95 (0.22)	0.63
TAT (WB) Volume	339.53 (237.71)	397.53 (236.26)	296.47 (229.48)	**< 0.01****
VAT (WB) SUV	0.95 (0.25)	0.92 (0.25)	0.97 (0.24)	**0.04***
VAT (WB) Volume	4017.02 (2373.72)	4638.99 (2440.92)	3559.96 (2214.4)	**< 0.01****
SKM (summed) SUV	0.8 (0.18)	0.78 (0.18)	0.82 (0.19)	**0.03***
SKM (summed) Volume	3546.06 (910.84)	3678.7 (804.27)	3448.86 (970.18)	0.06
SKM (L1-L5) SUV	0.75 (0.17)	0.73 (0.17)	0.77 (0.17)	**0.02***
SKM (L1-L5) Volume	2686.96 (679.75)	2701.8 (710.71)	2676.01 (655.75)	0.61
SAT (L1-L5) SUV	0.48 (0.15)	0.45 (0.13)	0.5 (0.16)	**< 0.01****
SAT (L1-L5) Volume	3759.43 (2318.02)	4387.36 (2516.42)	3296.05 (2039.55)	**< 0.01****
VAT (WB) SUV	0.93 (0.25)	0.89 (0.24)	0.97 (0.26)	**< 0.01****
VAT (WB) Volume	2627.12 (1536.75)	3002.82 (1553.48)	2349.88 (1463.7)	**< 0.01****
IMAT (L1-L5) SUV	0.69 (0.14)	0.67 (0.15)	0.76 (0.14)	**0.04***
IMAT (L1-L5) Volume	186.97 (159.19)	212.17 (158.78)	168.37 (158.01)	**< 0.01****

This table presents the clinical, imaging, and body composition characteristics of 253 patients, categorized by low weight loss (LoWL) and high weight loss (HiWL) groups. Categorical variables are shown as absolute numbers and percentages, while continuous variables are reported as median values with standard deviations (SD). Abbreviations: SKM, Skeletal Muscle; SAT, Subcutaneous Adipose Tissue; TAT, Thoracic Adipose Tissue; VAT, Visceral Adipose Tissue; IMAT, Intramuscular Adipose Tissue; AJCC, American Joint Committee on Cancer; HPV, Human Papillomavirus; CUP, Cancer of Unknown Primary; L1-L5, Lumbar Vertebra section L1-L5. *$p < 0.05$, **$p < 0.01$.

Table S2. Univariate and Multivariate Cox Regression Analysis of Covariates on Overall Survival.

Covariate	Univariate HR (95% CI)	p-Value	Multivariate HR (95% CI)	p-Value
WLGS	1.229 (1.059-1.427)	0.007**	0.908 (0.773-1.067)	0.242
Sex	0.856 (0.58-1.266)	0.437		
Age	1.022 (1.006-1.037)	0.007**	0.997 (0.977-1.018)	0.794
CUP	0.537 (0.132-2.181)	0.385		
Hypopharynx	2.32 (1.41-3.817)	0.001**	1.803 (0.94-3.455)	0.076
Larynx	1.125 (0.657-1.927)	0.667		
Nasopharynx	1.232 (0.392-3.875)	0.721		
Oral Cavity	1.664 (0.998-2.775)	0.051		
Oropharynx	0.59 (0.419-0.83)	0.002**	0.801 (0.514-1.247)	0.325
Stage	1.051 (0.869-1.272)	0.606		
HPV	0.361 (0.215-0.604)	< 0.001***	1.044 (0.559-1.951)	0.892
Smoking	1.937 (1.289-2.91)	0.001**	1.007 (0.624-1.625)	0.978
Feeding tube	1.145 (0.82-1.597)	0.427		
Tumor SUV	1.062 (0.996-1.132)	0.065		
Tumor Volume	1.019 (1.013-1.025)	< 0.001***	1.016 (1.009-1.023)	**< 0.001*****
Adrenal Glands SUV	2.148 (1.608-2.869)	< 0.001***	1.514 (0.626-3.66)	0.357
Adrenal Glands Volume	1.022 (0.973-1.074)	0.387		
Aorta SUV	1.791 (1.286-2.496)	0.001**	0.506 (0.174-1.467)	0.210
Aorta Volume	1.001 (0.998-1.003)	0.541		
GI Tract SUV	2.027 (1.309-3.14)	0.002**	1.109 (0.482-2.55)	0.808

GI Tract Volume	1.0 (0.999-1.0)	0.028*	1.0 (1.0-1.001)	0.277
Kidneys SUV	0.874 (0.679-1.125)	0.294		
Kidneys Volume	0.999 (0.997-1.001)	0.195		
Liver SUV	1.711 (1.227-2.386)	0.002**	1.735 (0.723-4.162)	0.217
Liver Volume	0.999 (0.999-1.0)	0.002**	1.001 (1.0-1.001)	0.114
Lungs SUV	0.057 (0.019-0.165)	< 0.001***	0.211 (0.029-1.544)	0.126
Lungs Volume	1.0 (1.0-1.0)	< 0.001***	1.0 (1.0-1.0)	0.847
Pancreas SUV	2.515 (1.777-3.56)	< 0.001***	3.033 (1.003-9.174)	**0.049***
Pancreas Volume	0.98 (0.974-0.987)	< 0.001***	0.984 (0.974-0.994)	**0.002****
Spine SUV	1.778 (1.187-2.664)	0.005**	1.064 (0.391-2.894)	0.904
Spine Volume	1.001 (1.0-1.002)	0.148		
Spleen SUV	1.795 (1.134-2.842)	0.013*	0.551 (0.18-1.685)	0.296
Spleen Volume	0.996 (0.995-0.998)	< 0.001***	0.999 (0.997-1.001)	0.239
Heart SUV	1.052 (0.895-1.236)	0.54		
Heart Volume	0.999 (0.998-1.0)	0.168		
SAT SUV	28.532 (11.704-69.559)	< 0.001***	1.46 (0.128-16.706)	0.761
SAT Volume	1.0 (1.0-1.0)	0.001**	1.0 (1.0-1.0)	**0.035***
SKM SUV	16.644 (7.321-37.84)	< 0.001***	18.211 (1.116-297.138)	0.042*
SKM Volume	1.0 (0.999-1.0)	0.014*	1.0 (0.999-1.0)	0.039*
VAT SUV	6.848 (3.862-12.142)	< 0.001***	0.239 (0.04-1.419)	0.115
VAT Volume	1.0 (1.0-1.0)	< 0.001***	1.0 (1.0-1.0)	0.719

Abbreviations: WLGS: Weight Loss Grading System; CUP: Cancer of Unknown Primary; HPV: Human Papillomavirus; SUV: Standardized Uptake Value; GI Tract: Gastrointestinal Tract; IMAT: Intramuscular Adipose Tissue; VAT: Visceral Adipose Tissue; SAT: Subcutaneous Adipose Tissue; SKM: Skeletal Muscle. *p < 0.05, **p < 0.01, ***p < 0.001.

Table S3. Model Performance Metrics Across Sites and Feature Sets.

Model/ Dataset	Accuracy (95% CI)	Sensitivity (95% CI)	Specificity (95% CI)	PPV (95% CI)	NPV (95% CI)	BACC (95% CI)	AUC (95% CI)
Composite / SUV+VOL	0.81 (0.80–0.82)	0.52 (0.48–0.55)	0.87 (0.86–0.88)	0.48 (0.45–0.51)	0.89 (0.89–0.90)	0.69 (0.68–0.71)	0.76 (0.74–0.78)
Composite All: Vienna_site	0.76 (0.74–0.76)	0.53 (0.50–0.57)	0.87 (0.85–0.88)	0.47 (0.44–0.50)	0.88 (0.87–0.89)	0.70 (0.68–0.72)	0.76 (0.74–0.76)
Composite All: TCIA_site	0.53 (0.51–0.55)	0.60 (0.58–0.62)	0.49 (0.47–0.51)	0.61 (0.59–0.63)	0.49 (0.47–0.50)	0.54 (0.53–0.56)	0.53 (0.51–0.55)
HiWL / SUV+VOL	0.56 (0.54–0.57)	0.60 (0.59–0.62)	0.49 (0.47–0.52)	0.61 (0.60–0.62)	0.48 (0.47–0.50)	0.55 (0.54–0.56)	0.60 (0.58–0.61)
Composite / SUV	0.79 (0.78–0.80)	0.53 (0.50–0.57)	0.84 (0.83–0.86)	0.43 (0.40–0.46)	0.89 (0.89–0.90)	0.69 (0.67–0.70)	0.75 (0.73–0.76)
HiWL / SUV	0.56 (0.55–0.57)	0.59 (0.57–0.61)	0.51 (0.49–0.53)	0.62 (0.60–0.63)	0.49 (0.48–0.50)	0.55 (0.54–0.57)	0.59 (0.57–0.60)
Composite / VOL	0.81 (0.79–0.82)	0.49 (0.46–0.53)	0.87 (0.86–0.88)	0.47 (0.44–0.50)	0.89 (0.88–0.90)	0.68 (0.66–0.70)	0.74 (0.72–0.76)
HiWL / VOL	0.56 (0.55–0.57)	0.59 (0.57–0.61)	0.52 (0.50–0.55)	0.62 (0.61–0.63)	0.49 (0.48–0.51)	0.56 (0.54–0.57)	0.60 (0.58–0.61)

This table presents the performance metrics of various machine learning models used to predict cachexia in head and neck squamous cell carcinoma (HNSCC) patients, including both composite endpoints of high weight loss (HiWL) combined with 1-year overall survival (OS) and HiWL alone. The table compares model accuracy, sensitivity, specificity, positive predictive value (PPV), negative predictive value (NPV), balanced accuracy (BACC), and area under the curve (AUC) across all datasets, as well as site-specific datasets from Vienna and TCIA. Abbreviations: SUV, Standardized Uptake Value; VOL, Volumetric Data; 95% CI, 95% Confidence Interval.
